# Exploratory Associations of Targeted Genetic Variants with Cephalometric Airway Parameters in Children with Skeletal Class II Sleep-Disordered Breathing Symptoms

**DOI:** 10.3390/children13030345

**Published:** 2026-02-27

**Authors:** Nazlı Karaca Kurt, Hilal Algul, Serdar Ceylaner, Gulay Ceylaner, Ayse Tuba Altug, Tulin Ufuk Toygar Memikoglu

**Affiliations:** 1 Private Orthodontic Practice, Istanbul 34394, Türkiye; 2 Department of Orthodontics, Faculty of Dentistry, Ankara University, Ankara 06560, Türkiye; 3 Intergen Genetic and Rare Diseases Diagnosis and Research Center, Ankara 06510, Türkiye

**Keywords:** cephalometric, airway analysis, genetic polymorphism, hyoid bone position, mandibular retrognathia, pediatric sleep-disordered breathing, respiratory event index, sleep-disordered breathing, skeletal class II malocclusion

## Abstract

**Highlights:**

**What are the main findings?**

- Targeted sequencing identified selected genetic variants in candidate pathways in a subset of children with skeletal Class II mandibular retrognathia and symptoms of sleep-disordered breathing.
- Variant carriers showed differences in cephalometric airway–related measures, most notably in hyoid-position–associated measurements.

**What are the implications of the main findings?**

- These exploratory data support integrated craniofacial and host-susceptibility phenotyping in pediatric sleep-disordered breathing and may help generate testable hypotheses for future studies.
- Larger controlled, ideally longitudinal cohorts with standardized phenotyping and appropriate multivariable analyses are required to validate genotype–phenotype patterns and clarify potential clinical relevance.

**Abstract:**

**Background/Objectives:** Pediatric sleep-disordered breathing (SDB) is influenced by craniofacial morphology and host susceptibility. Evidence integrating cephalometric airway features with targeted genetic variation in symptomatic skeletal Class II children remains limited. We explored whether children with skeletal Class II mandibular retrognathia and SDB symptoms harbor selected genetic variants and whether carriers show distinct cephalometric airway characteristics. **Methods:** This cross-sectional study included 48 children with skeletal Class II malocclusion, mandibular retrognathia, and snoring/mouth-breathing symptoms. Craniofacial and airway parameters were assessed on lateral cephalograms. SDB burden was evaluated by a baseline home sleep study (respiratory event index, REI). Targeted sequencing screened TNFRSF1A, PSTPIP1, SLC6A4 (5HTT), ACE, APOE, IRS1, and additionally PHOX2B and PMP22. Exploratory group comparisons used Student’s *t*-test. **Results:** Variants were identified in 13/48 participants (27%) in TNFRSF1A, PSTPIP1, SLC6A4, ACE, APOE, and IRS1; none were detected in PHOX2B or PMP22. C3–H was higher in variant carriers (39.90 ± 6.40 vs. 36.48 ± 3.95 mm; *p* < 0.05). HH1 (perpendicular distance from the hyoid bone to the C3–RGN line) was higher but not significant (16.99 ± 7.58 vs. 14.61 ± 5.25 mm; *p* > 0.05). **Conclusions:** In this clinically screened pediatric skeletal Class II cohort with SDB symptoms, selected genetic variants co-occurred with specific hyoid–cervical cephalometric features. Given the cross-sectional design, absence of a control group, and small number of carriers, findings are exploratory and require replication in larger, controlled cohorts with standardized phenotyping.

## 1. Introduction

Obstructive sleep apnea syndrome (OSAS) represents the most clinically consequential form of sleep-disordered breathing and is characterized by recurrent episodes of complete (apnea) or partial (hypopnea) upper-airway obstruction during sleep, commonly accompanied by oxygen desaturation and arousals [1]. OSAS is a multifactorial disorder arising from interactions between genetic susceptibility and environmental influences, with established risk determinants including craniofacial morphology, upper-airway soft-tissue and neuromuscular characteristics, elevated body mass index (BMI), and syndromic conditions [2,3].

In children, OSAS may occur from infancy through adolescence and typically shows no marked sex predilection [3]. Reported prevalence ranges from 1% to 5%, and higher rates among children with a family history of sleep-disordered breathing support a hereditary contribution [3]. Family-based investigations, including the Cleveland Family Study, further demonstrate familial clustering of OSAS [4]. In addition, the overrepresentation of OSAS within genetic syndromes such as trisomy 21, Fragile X syndrome, and Prader–Willi syndrome underscores the contribution of genetic factors to disease susceptibility.

Candidate genes implicated in OSAS pathophysiology include 5-HTT (SLC6A4), APOE, TNFRSF1A, ACE, IRS1, PHOX2B, PMP22, and PSTPIP1 [5,6,7,8]. Variants in these pathways may plausibly modulate upper-airway anatomy and collapsibility through effects on neuromuscular control, inflammatory signaling, and metabolic regulation. Nevertheless, most genetic evidence derives from adult cohorts, and pediatric data—particularly studies integrating genetic findings with craniofacial morphology—remain limited [9,10]. Accordingly, these genes were selected for hypothesis-driven targeted screening to explore potential genotype–phenotype co-occurrence patterns within a clinically characterized pediatric skeletal Class II cohort.

Craniofacial characteristics such as mandibular retrognathia and skeletal Class II malocclusion are well-recognized contributors to airway narrowing and functional obstruction. Lateral cephalometric analysis remains a widely used clinical adjunct for evaluating skeletal pattern and upper-airway dimensions, providing accessible craniofacial and airway-related information relevant to phenotyping [11]. However, integrative evidence combining cephalometric airway parameters with targeted genetic variation in symptomatic children is scarce.

Given that pediatric sleep-disordered breathing may persist across developmental stages, improved characterization of craniofacial and host susceptibility factors may help inform future work on phenotyping and potential risk pathways. We hypothesized that, within a clinically characterized skeletal Class II mandibular retrognathia cohort presenting with sleep-disordered breathing symptoms, the presence of selected genetic variants may co-occur with specific cephalometric airway characteristics. Therefore, this study aimed to explore variants identified by a candidate-gene panel in children with mandibular retrognathia and skeletal Class II malocclusion and to examine their associations with cephalometric airway features in an exploratory, hypothesis-generating framework.

## 2. Materials and Methods

### 2.1. Study Design and Ethical Approval

This cross-sectional study was approved by the Research Ethics Committee of the Faculty of Dentistry, Ankara University (Decision No: B.30.2.ANK.0.2.63.00/824-02/9-8/152; 12 December 2011) and was conducted in accordance with the Declaration of Helsinki. All participants and their parents/legal guardians were informed about the study procedures, including peripheral blood sampling for genetic testing and home-based sleep monitoring. Written informed consent was obtained from both the children and their legal guardians, including consent for the publication of anonymized data and images. The study is reported in accordance with the STROBE (Strengthening the Reporting of Observational Studies in Epidemiology) guidelines for cross-sectional studies.

This exploratory observational study did not include a non–Class II and/or asymptomatic control group. Accordingly, analyses were designed to describe within-cohort associations and are not intended to support phenotype specificity, diagnostic claims, or risk stratification.

### 2.2. Participants

The study included 48 children (21 boys and 27 girls) who presented to the Department of Orthodontics, Faculty of Dentistry, Ankara University, with mandibular retrognathia, skeletal Class II malocclusion, and clinically identified respiratory symptoms. All participants were free of syndromic conditions.

Inclusion criteria were: adequate physical and cognitive ability to comply with examinations; a history of frequent snoring during sleep and habitual mouth breathing; mandibular retrusion and an unfavorable soft-tissue profile; and being in an active growth and development period. Participants were required to have no systemic disease and no history of pharyngeal surgery.

Upper-airway phenotyping included a baseline otolaryngology consultation performed by an ENT specialist and documented using a standardized consultation form. The assessment covered the oropharynx and nasal cavity; tonsillar size was graded using the Brodsky scale. Children with clinically significant adenotonsillar hypertrophy (e.g., Brodsky grade 3–4) and/or relevant nasal obstruction findings were excluded.

The mean chronological age and sex distribution are summarized in Table 1. Sex, as a biological variable, was recorded as male or female based on sex assigned at birth as documented in the clinical records.

### 2.3. Data Collection and Measurements

Baseline records comprised lateral cephalometric and panoramic radiographs, dental casts, intraoral and extraoral photographs, hand–wrist radiographs, and home sleep study (HSS) recordings.

Craniofacial measurements were obtained from lateral cephalometric radiographs acquired using a Sirona Orthophos XG Plus Ds/Ceph unit (Sirona Dental Systems GmbH, Bensheim, Germany). Digitization and cephalometric analyses were performed using PORDIOS software (Purpose on Request Digitizer Input Output System; Institute for Orthodontic Computer Sciences, Aarhus, Denmark). Posterior pharyngeal airway dimensions were assessed according to the method described by Battagel and L’Estrange [11].

**Figure 1:**
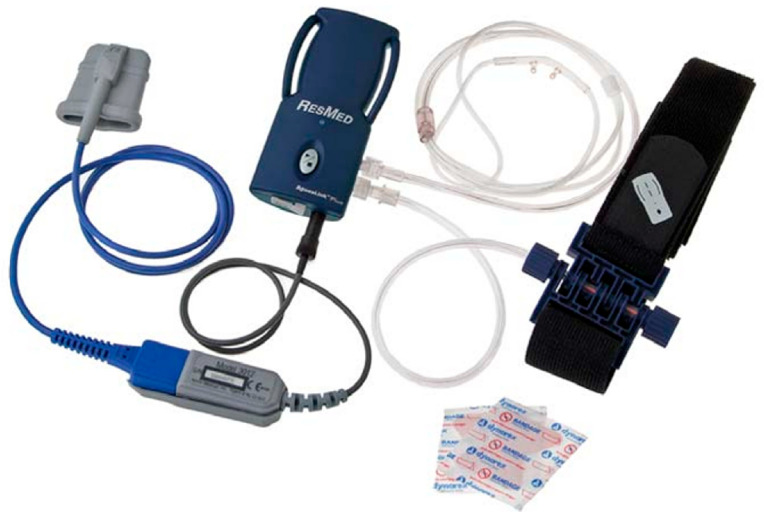
RESMED ApneaLink (ResMed Corp., San Diego, CA, USA) portable device used for home sleep monitoring.

**Figure 2:**
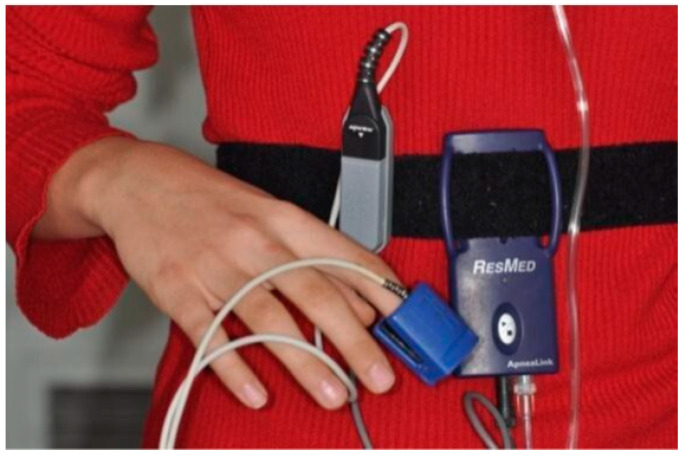
Portable home sleep monitoring device and components used in the study.

Respiratory function was evaluated using HSS recordings obtained with a portable sleep monitor (ApneaLink, ResMed Corp., San Diego, CA, USA) (Figure 1 and Figure 2). Data were transferred to a computer, automatically scored, and subsequently manually verified by N.K.K. using pediatric-specific scoring criteria. Because home monitoring does not provide polysomnography (PSG)-based sleep staging, the respiratory event index (REI), calculated from recording time, is not directly interchangeable with PSG-derived AHI in children and may underestimate severity. Accordingly, REI was used to describe respiratory event burden within an exploratory framework rather than to establish a PSG-equivalent diagnosis [12]. For transparency, a pragmatic threshold of REI > 1 event/hour was used to indicate evidence of sleep-disordered breathing on home monitoring, aligned with commonly used pediatric PSG thresholds in which AHI ≥ 1 event/hour is considered abnormal, while acknowledging that clinical cutoffs and severity definitions vary across pediatric literature [10]. Apnea and hypopnea measures are reported as total event counts (n) over the recording period, whereas REI is reported as events per hour.

Overnight attended polysomnography (PSG) is considered the reference standard for diagnosing pediatric obstructive sleep apnea; however, access to pediatric PSG is limited in many settings and may impose a substantial logistical burden on families. Accordingly, home-based cardiorespiratory sleep monitoring was used to characterize respiratory event burden in this clinically screened cohort. The respiratory event index (REI) derived from home monitoring was interpreted as an estimate rather than a PSG-equivalent diagnostic metric, and REI-based severity inferences were therefore made cautiously and limited to descriptive phenotyping. These data were integrated with craniofacial and genetic measures within an exploratory framework [12,13,14,15].

### 2.4. Variant Annotation and Interpretation

Detected sequence variants were annotated and interpreted using a stepwise workflow. First, each variant was screened against reference databases and the literature (Human Gene Mutation Database (HGMD), NCBI dbSNP, and PubMed) to determine whether it had been previously reported. Variants not catalogued in these resources were further evaluated using in silico prediction tools (SIFT4G v2.0.0, PROVEAN v1.1.5, MutationTaster2, and Human Splicing Finder (HSF) v3.1) to estimate potential functional consequences, including missense effects and splice-related disruption. During laboratory interpretation, population reference datasets (e.g., 1000 Genomes and ExAC) were consulted to contextualize whether detected variants were commonly observed in reference populations; however, numerical allele-frequency values were not extracted for reporting in the present study.

This annotation strategy was implemented to support exploratory genotype–phenotype comparisons in a clinically screened cohort. Accordingly, we use the term “variant” throughout and do not imply clinical pathogenicity. Formal clinical classification under the ACMG/AMP framework and functional validation were beyond the scope of this work. Therefore, the genetic findings should be interpreted as descriptive and hypothesis-generating rather than as evidence of clinically established pathogenic variants.

### 2.5. Genetic Analysis

Peripheral blood samples were collected from 48 pediatric participants and 61 parents into EDTA-containing tubes. Genomic DNA was extracted from 200 μL peripheral blood using the QIAamp DNA Blood Mini Kit (QIAGEN GmbH, Hilden, Germany) and stored at −20 °C until processing. DNA concentration was measured by NanoDrop ND-1000 spectrophotometer (Thermo Fisher Scientific Inc., Waltham, MA, USA), and integrity was assessed by 2% agarose gel electrophoresis. All genetic procedures were conducted at the Intergen Genetic Diagnosis Research and Application Center.

Targeted amplicon-based sequencing was performed to screen TNFRSF1A, PSTPIP1, SLC6A4 (5HTT), ACE, APOE, IRS1, PHOX2B, and PMP22. PCR primers were designed to cover coding exons and exon–intron junctions. The candidate-gene panel was predefined based on prior literature implicating these genes in sleep-disordered breathing/OSAS susceptibility and/or biologic pathways plausibly linked to pediatric upper-airway collapsibility, including inflammatory signaling (TNFRSF1A, PSTPIP1), neuromodulation/serotonergic tone (SLC6A4), metabolic/vascular regulation (ACE, APOE, IRS1), and ventilatory control/neuromuscular mechanisms (PHOX2B, PMP22). Given the exploratory aim, the genetic component was intended for descriptive variant screening and hypothesis generation rather than formal gene-level association testing or clinical pathogenicity inference. PCR reaction composition and thermal cycling conditions (standard and long PCR) are provided in Supplementary Tables S1 and S2, and the targeted regions with expected amplicon sizes are summarized in Supplementary Table S3.

To optimize sequencing efficiency, participant DNA samples were pooled into six pools (eight samples per pool). Pooling volumes were adjusted inversely proportional to DNA concentration to promote balanced representation within each pool. PCR products were combined considering amplification yield and amplicon size to generate homogeneous pools for downstream sequencing. Amplified products were purified using the NucleoFast® 96 PCR clean-up kit (MACHEREY-NAGEL GmbH & Co. KG, Düren, Germany) quantified and standardized to 0.2 ng/μL.

Sequencing libraries were prepared from purified PCR pools using the Nextera XT sample preparation kit (Illumina, Inc., San Diego, CA, USA) and sequenced on an Illumina MiSeq platform using MiSeq Reagent Kit v2 (2 × 150 bp; Illumina Inc.) according to the manufacturer’s protocol. Sequence data were inspected using Integrative Genomics Viewer (IGV) v2.3. Because pooled sequencing was employed, the minimum variant allele fraction threshold was set at 0.02 to enable detection of low-frequency signals consistent with a single variant allele within a pooled sample set. When a variant signal was detected in a pool, the corresponding target region was re-amplified and re-sequenced in individual samples to identify the carrier(s) and confirm the finding. Orthogonal confirmation using an independent method (e.g., Sanger sequencing) was not performed and is acknowledged as a limitation.

Variants are reported using standard nomenclature and are referred to as “variants” rather than “mutations” unless pathogenicity is supported by external evidence. Parental samples were collected to support confirmation when required; however, formal segregation or de novo analyses were not conducted and are acknowledged as additional limitations.

### 2.6. Statistical Analysis

A total of 48 parameters derived from lateral cephalometric radiographs and portable sleep monitoring were included in the statistical analysis. Method error was assessed using intraclass correlation coefficients (ICC) with 95% confidence intervals. Intra-observer reliability was excellent overall (ICC ≈ 0.90–1.00), with the great majority of measurements exceeding 0.95, indicating high repeatability of cephalometric and airway variables. Variable-specific ICCs (95% CIs) are reported in Supplementary Table S4.

Descriptive statistics were computed for all cephalometric, airway, and sleep study measures. Between-group comparisons were performed using Student’s *t*-test to compare participants without identified genetic variants (non-carriers, n = 35) and those with identified variants (carriers, n = 13). Given the number of outcomes assessed and the limited sample size, analyses were considered exploratory; therefore, nominal two-sided *p*-values are reported with the explicit caution that unadjusted multiple comparisons increase the likelihood of false-positive findings. Accordingly, statistically significant results were interpreted as hypothesis-generating and not as evidence of diagnostic or predictive utility. Comparisons within the very small subgroup carrying variants localized to the same gene region (n = 3) were treated as descriptive only due to insufficient statistical power. Statistical significance was set at *p* < 0.05 (two-sided) for reporting purposes.

### 2.7. Clinical Trial Registration

This study was designed as a cross-sectional observational study and did not involve any prospective intervention.

## 3. Results

### 3.1. Skeletal and Airway Measurements

Given the cross-sectional design, results are reported as descriptive associations and do not imply temporality, causality, or predictive utility. Cephalometric skeletal and airway variables are compared between participants without identified genetic variants (non-carriers, n = 35) and those with identified variants (carriers, n = 13) in Table 2 and Table 3. Because analyses were exploratory and multiple outcomes were assessed, statistically significant findings are interpreted cautiously and are considered hypothesis-generating. Comparisons within the very small subgroup carrying variants localized to the same gene region (n = 3) are presented for descriptive purposes only.

Among skeletal linear measurements, Cd–VER, Cd–A, and Go–Gn were higher in variant carriers than in non-carriers (Cd–VER: 15.59 ± 2.77 vs. 11.96 ± 4.47; Cd–A: 93.18 ± 5.67 vs. 88.91 ± 5.76; Go–Gn: 73.38 ± 6.76 vs. 69.38 ± 4.54; Table 2; *p* < 0.05 for Cd–VER and Go–Gn, and *p* < 0.01 for Cd–A). For airway- and hyoid-related measures, HH1 (perpendicular distance from the hyoid bone to the C3–RGN line) was higher in carriers but did not reach statistical significance (16.99 ± 7.58 vs. 14.61 ± 5.25; *p* > 0.05), whereas C3–H was significantly higher in carriers (39.90 ± 6.40 vs. 36.48 ± 3.95; *p* < 0.05) (Table 3).

### 3.2. Home Sleep Apnea Testing (HSAT) and Body Mass Index (BMI)

HSAT-derived respiratory indices and oxygenation metrics are summarized in Table 4. No statistically significant between-group differences were observed between variant carriers (n = 13) and non-carriers (n = 35) in REI, apnea/hypopnea event counts, ODI, or oxygen saturation parameters (all *p* > 0.05). In contrast, BMI was higher in variant carriers than in non-carriers (20.64 ± 2.95 vs. 18.31 ± 3.21 kg/m^2^; *p* < 0.05) (Table 5).

### 3.3. Genetic Findings

Targeted sequencing was performed for TNFRSF1A, PSTPIP1, SLC6A4 (5HTT), ACE, APOE, IRS1, PHOX2B, and PMP22. No variants were detected in PHOX2B or PMP22. Overall, variants were identified in 13 of 48 participants (27%), representing 13 distinct alterations across six genes (ACE, APOE, SLC6A4, IRS1, TNFRSF1A, and PSTPIP1) (Table 6). Among variant carriers, six were female and seven were male.

Variants were distributed across six genes. In SLC6A4, one participant carried p.G56A; in APOE, one participant carried p.I166del; and in TNFRSF1A, one participant carried p.R121Q. ACE harbored five variants, including p.Y244C (c.731A>G) in three participants, and p.G354R (c.1060G>A), p.R570Q (c.1709G>A), p.R778W (c.2332C>T), and p.R1279Q (c.3876G>A) each in one participant. In IRS1, three variants were detected (p.K161K, p.G597E, and p.P912L); notably, the participant carrying IRS1 p.K161K also harbored ACE p.R778W. In PSTPIP1, two variants (p.G403R and p.R405C) were identified, each in a different participant.

## 4. Discussion

Obstructive sleep apnea syndrome (OSAS) is a major public health concern with substantial effects on quality of life and long-term health outcomes. Beyond recurrent apneas and hypopneas, OSAS is associated with excessive daytime sleepiness, snoring, morning headaches, cognitive impairment, cardiovascular complications, and increased mortality risk [16]. In children, OSAS differs from adult disease in etiology, prevalence, and clinical course, with peak incidence reported between 2 and 8 years of age [15]. Polysomnography-based prevalence estimates range from 1% to 3%, with higher values reported in selected populations [17], and affected children may exhibit impaired growth, reduced academic performance, and cognitive deficits [18].

Craniofacial morphology is a well-established contributor to pediatric sleep-disordered breathing. Children with skeletal Class II malocclusion—particularly those with mandibular retrognathia and a convex profile—have been shown to exhibit increased upper airway obstruction on sagittal cephalometric evaluation [19]. In addition to skeletal factors, soft tissue characteristics, neck circumference, and body mass index (BMI) are recognized determinants of disease burden. In the present cohort, BMI was higher in variant carriers than in non-carriers (20.64 ± 2.95 vs. 18.31 ± 3.21 kg/m^2^; *p* < 0.05), raising the possibility of confounding or mediation in observed between-group differences. Accordingly, the genotype–cephalometric co-occurrences reported here should not be interpreted as independent genetic effects; rather, they represent descriptive associations within a clinically screened sample that may be influenced by unmodeled covariates (including BMI, age, sex, and pubertal status). Increasing evidence supports a genetic contribution to pediatric OSAS; however, pediatric data integrating craniofacial phenotyping with targeted genetic variation remain limited [20].

In this study, we explored whether selected genetic variants co-occur with cephalometric airway characteristics in children with skeletal Class II malocclusion and mandibular retrognathia presenting with sleep-disordered breathing symptoms. Although this integrative approach is clinically appealing, the absence of a control group without skeletal Class II malocclusion and/or without sleep-disordered breathing constrains interpretation. Therefore, the findings should be viewed as exploratory within-cohort associations rather than evidence of diagnostic specificity or population-level risk stratification.

Hyoid bone position is a key determinant of tongue posture and airway patency. Inferior displacement of the hyoid has been reported in OSAS and may reflect compensatory adaptation and/or altered neuromuscular control. HH1—defined here as the perpendicular distance from the hyoid bone to the C3–RGN reference line—reflects the superoinferior (vertical) position of the hyoid within the mandibular–cervical framework and has been associated with OSAS severity in prior studies, with larger values reported in affected individuals [21]. In the present cohort, HH1 was higher in variant carriers but did not reach statistical significance, suggesting that any HH1-related tendency may be subtle and sample-size dependent. Similarly, MP–H, which reflects the vertical relationship between the mandibular plane and the hyoid, has been reported to increase in OSAS [22]. MP–H did not differ significantly between carriers and non-carriers; however, in the very small subgroup with variants localized to the same gene region (n = 3), MP–H showed a higher mean value, warranting confirmation in larger samples.

Previous studies indicate that individuals with skeletal Class II malocclusion frequently exhibit a narrowed pharyngeal airway and altered hyoid position relative to the mandible and cervical vertebrae [23,24]. Consistent with this concept, the subgroup with variants localized to the same gene region (n = 3) demonstrated a shorter C3–RGN distance and a greater H–RGN distance compared with non-carriers, descriptively suggesting an altered hyoid–mandibular–cervical relationship. However, given the very small subgroup size, these patterns should be interpreted cautiously and considered hypothesis-generating. Reduced inferior airway space has also been reported to correlate negatively with apnea–hypopnea index values [23]. Although home sleep apnea testing was used here and respiratory event index (REI) was evaluated instead of polysomnography-derived AHI, the craniofacial–airway relationships remain conceptually comparable, while acknowledging that HSAT-derived indices are not fully interchangeable with PSG-derived measures in pediatric populations. Accordingly, severity-related interpretations were kept cautious and framed as exploratory [12].

The genetic findings further support the multifactorial nature of pediatric sleep-disordered breathing. Variants were identified in genes with plausible links to inflammatory pathways (TNFRSF1A, PSTPIP1), neuromodulation (SLC6A4), metabolic/vascular regulation (ACE, APOE), and insulin signaling (IRS1) [24,25,26,27,28,29,30,31,32]. Although PHOX2B and PMP22 have been implicated in ventilatory control and OSAS susceptibility [29], no variants were detected in these genes in the present cohort. Synonymous variants (e.g., IRS1 p.K161K) were reported descriptively, and their functional relevance remains uncertain unless supported by splicing or regulatory evidence. More broadly, several detected variants involved amino-acid changes in genes with biologically plausible links to sleep-disordered breathing pathways; nevertheless, the current dataset does not allow inference of clinical pathogenicity.

Variants in TNFRSF1A and PSTPIP1, which are associated with autoinflammatory conditions, may be relevant given the established role of pro-inflammatory cytokines—including tumor necrosis factor-α—in upper airway pathology [25,26,27]. Serotonergic pathways also play a key role in maintaining upper airway tone during sleep [30,31,32,33], and the identification of an SLC6A4 variant aligns with prior reports linking serotonergic gene variation to OSAS susceptibility. ACE variants were observed in six participants, including the Y244C polymorphism and additional rare variants. Prior work on ACE polymorphisms in OSAS has been inconsistent [34,35,36,37]; however, altered ACE activity may plausibly contribute to hypoxia-related vascular and inflammatory responses. A rare ACE variant (R778W) was also observed in this cohort, but in the absence of functional validation and quantitative population-frequency annotation, its clinical significance should be interpreted cautiously. Additional variants in APOE and IRS1 may influence respiratory hemodynamics and skeletal muscle function, respectively, and could modulate vulnerability to airway obstruction [33,34,35,36,37,38,39,40,41,42,43].

Several design features necessarily temper interpretation. Multiple cephalometric and airway outcomes were examined in a relatively small cohort, and unadjusted comparisons increase the possibility of chance findings. Moreover, the small number of variant carriers (n = 13)—and especially the very small exploratory subgroup analyses (n = 3)—substantially limits statistical power and makes genotype–phenotype inference tentative. Therefore, the present results should be interpreted as descriptive and hypothesis-generating. From a clinical perspective, these observations may help generate testable hypotheses regarding craniofacial–airway phenotypes and host susceptibility factors in pediatric sleep-disordered breathing; however, the cross-sectional design precludes temporality or prediction, and replication in larger, controlled cohorts with standardized phenotyping remains essential.

### Limitations and Future Directions

This study has several limitations. First, the absence of a non–skeletal Class II and/or asymptomatic control group (or an ethnically matched comparator) restricts the interpretation of phenotype specificity and prevents evaluating variant frequencies as enrichment; therefore, the findings should be interpreted as within-cohort, exploratory associations rather than population-level inference. In addition, only 13 participants were variant carriers, and exploratory subgroup comparisons relied on very small numbers (n = 3), which markedly reduces statistical power and increases uncertainty around genotype–phenotype patterns. The single-center, cross-sectional design limits generalizability and precludes causal inference or assessment of temporality. Moreover, the number of outcomes assessed relative to sample size increases the likelihood of chance findings in univariable comparisons.

Second, home sleep monitoring was used because access to pediatric polysomnography (PSG) is limited in many settings; however, portable systems typically do not provide full neurophysiologic sleep staging or arousal scoring and may therefore underestimate event indices compared with attended PSG [12]. Accordingly, REI was used for exploratory phenotyping rather than definitive diagnosis, and this constraint should be considered when interpreting associations with craniofacial and genetic measures. Craniofacial assessment was based on two-dimensional cephalometric radiographs rather than three-dimensional imaging.

Third, genetic analysis was restricted to a targeted candidate-gene panel; thus, several identified variants were rare and require independent validation, including segregation/inheritance assessment and functional interpretation. We did not assign ACMG/AMP pathogenicity categories, report numerical population allele frequencies, or perform orthogonal validation; therefore, genetic findings should be regarded as descriptive. Potential confounders—including age, sex, BMI (which differed significantly between groups), and pubertal status—were not incorporated into multivariable models; thus, the reported associations should not be interpreted as independent genetic or craniofacial effects.

Finally, the exclusive inclusion of Turkish pediatric participants limits extrapolation to other populations. Future multicenter studies with larger, controlled, and longitudinal cohorts, standardized phenotyping (including ENT evaluation and pubertal staging), and prespecified primary outcomes with multivariable and multiplicity-aware analytical strategies are warranted.

## 5. Conclusions

In this clinically characterized pediatric skeletal Class II cohort with mandibular retrognathia and symptoms of snoring and mouth breathing, selected candidate-gene variants co-occurred with specific cephalometric airway–related features, particularly hyoid-position measures. Given the cross-sectional design, absence of a control group, limited number of variant carriers, and use of home sleep monitoring (REI), these findings are best interpreted as descriptive and hypothesis-generating rather than diagnostically or causally informative. Larger controlled, ideally longitudinal studies with standardized phenotyping, multivariable modeling, and—where feasible—segregation and functional validation are needed to determine whether combined craniofacial and genetic markers have clinical utility in pediatric sleep-disordered breathing.

## Figures and Tables

**Table 1:** Sex distribution and chronological age of participants (*n* = 48).

Group	Class II (*n* = 48; 27 Females, 21 Males)
Chronological Age (years) Mean ± SD	11.17 ± 1.87
Min.	7.41
Max.	16.5

SD: Standard deviation. Min: Minimum value; Max: Maximum value.

**Table 2:** Comparison of skeletal cephalometric variables between participants without identified genetic variants (non-carriers) and those with identified genetic variants (carriers).

Skeletal Angular Measurements	Group Without Identified Genetic Variants (*n* = 35) Mean ± SD	Group with Identified Genetic Variants (*n* = 13) Mean ± SD	*p*-Value (Student t)	*p*-Value
SNA	79.82 ± 3.28	79.70 ± 2.66	0.907	Ns
SNB	72.60 ± 2.67	73.09 ± 3.32	0.600	Ns
ANB	7.22 ± 1.75	6.61 ± 1.98	0.306	Ns
SN/MP	37.18 ± 6.42	34.58 ± 7.30	0.236	Ns
PP/MP	29.12 ± 6.93	27.38 ± 6.92	0.443	Ns
A-VER	75.67 ± 3.02	75.84 ± 4.56	0.881	Ns
A-HOR	77.82 ± 2.89	78.02 ± 4.80	0.861	Ns
B-VER	69.10 ± 4.04	68.26 ± 4.55	0.539	Ns
B-HOR	103.97 ± 4.79	104.28 ± 7.81	0.869	Ns
Gn-VER	69.74 ± 4.17	69.34 ± 5.60	0.789	Ns
Gn-HOR	116.02 ± 5.38	117.81 ± 9.06	0.404	Ns
Go-VER	6.13 ± 3.54	4.19 ± 2.87	0.084	Ns
Go-HOR	54.38 ± 4.16	52.37 ± 5.40	0.177	Ns
Cd-VER	11.96 ± 4.47	15.59 ± 2.77	0.009	**
Cd-HOR	9.15 ± 3.73	6.89 ± 3.63	0.067	Ns
N-ANS	52.87 ± 2.52	53.48 ± 3.38	0.501	Ns
ANS-Me	68.97 ± 5.90	68.79 ± 6.93	0.929	Ns
N-Me	118.95 ± 7.55	119.36 ± 9.38	0.876	Ns
S-Go	73.31 ± 5.99	74.46 ± 7.61	0.586	Ns
Cd-A	88.91 ± 5.76	93.18 ± 5.67	0.027	*
Cd-Gn	107.52 ± 6.75	111.47 ± 8.40	0.099	Ns
Cd-Go	50.97 ± 5.49	51.59 ± 6.54	0.743	Ns
Go-Gn	69.38 ± 4.54	73.38 ± 6.76	0.022	*

SD: Standard Deviation; *p*-values are reported using significance bands (NS, *, **) *p* < 0.05 * *p* < 0.01 ** Ns: non significant; Angular measurements are given in degrees (°) and linear measurements in millimeters (mm). SNA, Sella–Nasion–A point angle; SNB, Sella–Nasion–B point angle; ANB, A point–Nasion–B point angle; SN/MP, angle between the Sella–Nasion (SN) plane and the mandibular plane (MP); PP/MP, angle between the palatal plane (PP; ANS–PNS) and MP (Go–Gn). A-VER/A-HOR, B-VER/B-HOR, Gn-VER/Gn-HOR, Go-VER/Go-HOR, Cd-VER/Cd-HOR indicate the vertical (VER) and horizontal (HOR) linear distances of Point A, Point B, Gnathion (Gn), Gonion (Go), and Condylion (Cd), respectively, relative to the cephalometric reference coordinate system used in this study. N-ANS, upper anterior facial height (Nasion–ANS); ANS-Me, lower anterior facial height (ANS–Menton); N-Me, total anterior facial height (Nasion–Menton); S-Go, posterior facial height (Sella–Gonion); Cd-A (Co-A), maxillary length; Cd-Gn (Co-Gn), mandibular length; Cd-Go (Co-Go), ramus height; Go-Gn, mandibular body length.

**Table 3:** Comparison of airway- and hyoid-related cephalometric variables between participants without identified genetic variants (non-carriers) and those with identified genetic variants (carriers).

Airway Dimensional Measurements	Group Without Identified Genetic Variants (*n* = 35) Mean ± SD	Group with Identified Genetic Variants (*n* = 13) Mean ± SD	*p*-Value (Student t)	*p*-Value
PNS-P	34.48 ± 4.37	34.83 ± 4.91	0.813	Ns
SPC-SPD	17.40 ± 2.09	17.50 ± 2.48	0.889	Ns
P-SPpp	25.58 ± 4.70	26.00 ± 4.64	0.784	Ns
PNS-PPW1	22.10 ± 4.03	24.22 ± 3.30	0.097	Ns
P-PPW2	9.56 ± 2.58	9.56 ± 2.52	0.999	Ns
Eb-PPW3	15.40 ± 3.33	14.74 ± 3.87	0.545	Ns
Eb-TT	72.01 ± 5.10	73.40 ± 5.60	0.412	Ns
PNS-Eb	57.96 ± 6.49	58.17 ± 7.46	0.920	Ns
MP-H	17.02 ± 6.00	17.27 ± 6.15	0.899	Ns
C3-RGN	62.24 ± 5.53	63.84 ± 5.43	0.375	Ns
HH1	14.61 ± 5.25	16.99 ± 7.58	0.224	Ns
H-RGN	33.17 ± 5.00	33.87 ± 4.56	0.661	Ns
C3-H	36.48 ± 3.95	39.90 ± 6.40	0.030	*

SD: Standard Deviation; *p*-values are reported using significance bands (NS, *) *p* < 0.05 * Ns: non significant; PNS–P, distance from posterior nasal spine (PNS) to posterior pharyngeal wall; SPC–SPD, soft palate length (soft palate contour length); P–SPpp, distance from posterior pharyngeal wall (P) to the soft palate at the palatal plane level (SPpp); PNS–PPW1, distance from PNS to posterior pharyngeal wall 1 (upper pharyngeal width); P–PPW2, distance from point P to posterior pharyngeal wall 2 (middle pharyngeal width); Eb–PPW3, distance from epiglottis base (Eb) to posterior pharyngeal wall 3 (lower pharyngeal width); Eb–TT, distance from epiglottis base (Eb) to tongue tip (TT); PNS–Eb, distance from PNS to epiglottis base (Eb); MP–H, perpendicular distance from the mandibular plane (MP; Go–Gn) to the hyoid bone (H); C3–RGN, linear distance between the third cervical vertebra point (C3) and retrognathion (RGN); HH1, perpendicular distance from the hyoid bone (H) to the C3–RGN reference line; H–RGN, distance from H to RGN; C3–H, distance from C3 to H.

**Table 4:** HSAT-derived respiratory event indices and oxygenation metrics in variant-carriers and non-carriers.

HSS Mean Values	Group Without Identified Genetic Variants (*n* = 35) Mean ± SD	Group with Identified Genetic Variants (*n* = 13) Mean ± SD	*p*-Value (Student t)	*p*-Value
REI (events/hour)	3.31 ± 1.69	2.77 ± 1.64	0.327	Ns
Apnea (n)	12.83 ± 8.25	11.15 ± 8.62	0.539	Ns
Hypopnea (n)	10.54 ± 10.71	5.46 ± 4.54	0.106	Ns
ODI (events/hour)	4.23 ± 3.63	3.31 ± 2.59	0.408	Ns
#DeSaO2 (%)	31.17 ± 26.63	21.08 ± 17.86	0.214	Ns
B-SaO2 (%)	98.20 ± 0.72	98.00 ± 0.58	0.374	Ns
Ort.SaO2 (%)	96.03 ± 0.89	95.92 ± 1.26	0.736	Ns
Ed-SaO2 (%)	88.50 ± 5.20	85.77 ± 7.30	0.156	Ns

SD: Standard Deviation, Ns: non-significant; Apnea (n) and Hypopnea (n) represent total event counts scored during the HSAT recording; REI (events/hour) represents the number of respiratory events per hour calculated using recording time (not PSG-derived sleep time); ODI: Oxygen Desaturation Index.

**Table 5:** Body mass index (BMI) by genetic variant status (non-carriers vs. carriers).

Parameters	Group Without Identified Genetic Variants (*n* = 35) Mean ± SD	Group with Identified Genetic Variants (*n* = 13) Mean ± SD	*p*-Value (Student t)	*p*-Value
BMI (kg/m^2^)	18.31 ± 3.21	20.64 ± 2.95	0.027	*

BMI: Body Mass Index; SD: Standard Deviation, *p* < 0.05 * Ns: non significant.

**Table 6:** Genetic variants identified in children with skeletal Class II malocclusion and sleep-disordered breathing symptoms.

Gene	Exon	Codon	Position	cDNA	Reference Allele (Ref)	Alternate Allele (Alt)	Protein Change	dbSNP/ID	Variant Consequence
TNFRSF1A	4	121	2	362	G	A	p.R121Q	rs4149584	Missense
PSTPIP1	15	403	1	1207	G	C	p.G403R	Not available	Missense
PSTPIP1	15	405	1	1213	C	T	p.R405C	rs201253322	Missense
SLC6A4 (5HTT)	3	56	2	167	G	C	p.G56A	CM057408	Missense
ACE	5	244	2	731	A	G	p.Y244C	rs3730025	Missense
ACE	7	354	1	1060	G	A	p.G354R	rs56394458	Missense
ACE	11	570	2	1709	G	A	p.R570Q	rs371599063	Missense
ACE	16	778	1	2332	C	T	p.R778W	Not available	Missense
ACE	25	1279	2	3836	G	A	p.R1279Q	rs200754517	Missense
APOE	4	166		c.496-498delCTC			p.166delL	CD002895	In-frame deletion
IRS1	1	161	3	483	A	G	p.K161K	Not available	Synonymous
IRS1	1	597	2	1790	G	A	p.G597E	Not available	Missense
IRS1	1	912	2	2735	C	T	p.P912L	Not available	Missense

ACE: Angiotensin-Converting Enzyme; APOE: Apolipoprotein E; RS1: İnsulin Receptor Substrate Variants are reported descriptively; clinical pathogenicity should not be inferred in the absence of formal classification and functional validation. Where available, dbSNP/ID is provided for traceability; however, no clinical pathogenicity classification is assigned in this study.

## Data Availability

The data presented in this study are not publicly available due to ethical and privacy restrictions related to human genetic and clinical data. The data supporting the findings of this study are available from the corresponding author upon reasonable request.

## Supplementary Materials

The following supporting information can be downloaded at: https://www.mdpi.com/article/10.3390/children13030345/s1.
