# Supplementary material for: Exploratory Associations of Targeted Genetic Variants with Cephalometric Airway Parameters in Children with Skeletal Class II Sleep-Disordered Breathing Symptoms
Source: Children (Basel). 2026 Feb 27;13(3):345. doi: 10.3390/children13030345 (PMC13025268; doi:10.3390/children13030345)
Supplement: Supplementary file 1 [file children-13-00345-s001.zip › children-4106072-supplementary.pdf]

Supplementary Table S1. PCR reaction mixture and thermal cycling conditions

PCR reaction components protocols used for standard and long PCR amplifications in the targeted amplicon-based sequencing workflow.

**Standard PCR**	
**Reaction components**	**Volume per reaction (µL)**
dH20	15
5x Buffer (Thermo Inc.)	5
dNTP mixture, each one 10mM	0,5
Forward Primer (5 µM)	1
Back Primer (5 µM)	1
PhireII HS DNA Polimeraz (Thermo Inc.)	0,5
Template DNA (20-50 ng/µl)	2
Total	25

**Long PCR**	
**Reaction components**	**Volume per reaction (µL)**
dH20	14,5
10x Buffer (Thermo Inc.)	2,5
dNTP mixture, each one 10mM	0,6
Forward Primer (5 µM)	2,5
Back Primer (5 µM)	2,5
SuperHotTaq DNA Polimeraz (Bioron Inc.)	0,4
Template DNA (20-50 ng/µl)	2
Total	25

Supplementary Table S2. PCR thermal cycling conditions

PCR thermal cycling protocols used for standard and long PCR amplifications in the targeted amplicon-based sequencing workflow.

**Standard PCR**		
**Temperature (°C)**	**Time (min:s)**	**Cycles**
95	10:00	1
95	00:45	35
60	00:45	35
72	00:45	35
72	10:00	1
4	∞	1
95	10:00	1
95	00:45	35
**Long PCR**		
**Temperature (°C)**	**Time (min:s)**	**Cycles**
94	3:00	1
94	00:20	10
60	00:30	30
68	10:00	30
94	00:20	30
60	00:30	30
68	10:00	30
	(+5 s/cycle)	
68	10:00	1

Supplementary Table S3. Targeted amplicon design and expected product sizes

Target genes, amplified exonic regions, and expected PCR product sizes (bp) for the amplicon-based sequencing design.

Genes	Exons	Length (bp)	PCR
PMP22	2	608	Standard
PMP22	3	512	Standard
PMP22	4	865	Standard
PMP22	5	640	Standard
PHOX2B	1	725	Standard
PHOX2B	2	436	Standard
PHOX2B	3	733	Standard
ACE	1-2	1392	Standard
ACE	3-7	3077	Standard
ACE	8-12	2843	Standard
ACE	13	1363	Standard
ACE	14-15	955	Standard
ACE	16-17	1554	Standard
ACE	18-19	693	Standard
ACE	20-22	1440	Standard
ACE	23-25	1530	Standard
APOE	2	373	Standard
APOE	3-4	1849	Standard
SLC6A4	2	967	Standard
SLC6A4	3-5	2150	Standard
SLC6A4	6-7	875	Standard
SLC6A4	8	494	Standard
SLC6A4	9-10	1302	Standard
SLC6A4	11	319	Standard
SLC6A4	12	853	Standard
SLC6A4	13	707	Standard
SLC6A4	14	318	Standard
IRS1	1	4297	Uzun

TNF	1-4	2171	Standard
TNFRSF1A	1	332	Standard
TNFRSF1A	2-3	716	Standard
TNFRSF1A	4-5	690	Standard
TNFRSF1A	6-7	542	Standard
TNFRSF1A	8-9	841	Standard
TNFRSF1A	10	523	Standard
PSTPIP1	1	435	Standard
PSTPIP1	2-3	613	Standard
PSTPIP1	4-5	596	Standard
PSTPIP1	6	294	Standard
PSTPIP1	7	401	Standard
PSTPIP1	8	327	Standard

Supplementary Table S4. Intra-observer reliability of cephalometric and airway measurements (ICC with 95% CI)”

**Skeletal Angular Measurements**	
**SNA**	99,41
**SNB**	99,50
**ANB**	97,30
**SN/MP**	99,32
**PP/MP**	99,63
**A-VER**	97,63
**A-HOR**	98,21
**B-VER**	98,04
**B-HOR**	98,75
**Gn-VER**	98,35
**Gn-HOR**	99,70
**Go-VER**	98,46
**Go-HOR**	97,31
**Cd-VER**	96,94
**Cd-HOR**	95,15

**N-ANS**	97,73
**ANS-Me**	99,59
**N-me**	99,78
**S-Go**	98,81
**Cd-A**	99,05
**Cd-Gn**	99,40
**Cd-Go**	97,93
**Go-Gn**	97,95
**Airway Dimensional Measurements**	
**PNS-P**	98,76
**SPC-SPD**	97,48
**P-SPpp**	98,83
**PNS-PPW1**	97,54
**P-PPW2**	98,52
**Eb-PPW3**	90,29
**Eb-TT**	98,47
**PNS-Eb**	99,50
**MP-H**	99,90
**C3-RGN**	99,53
**HH1**	99,47
**H-RGN**	99,42
**C3-H**	99,68
